# Correction: Exploring the antifouling effect of elastic deformation by DEM–CFD coupling simulation

**DOI:** 10.1039/d0ra90112a

**Published:** 2020-11-11

**Authors:** Limei Tian, E. Jin, Jianfu Wang, Xiaoming Wang, Wei Bing, Huichao Jin, Jie Zhao, Luquan Ren

**Affiliations:** Key Laboratory of Bionic Engineering (Ministry of Education), Jilin University No. 5988 Renmin Street Changchun 130022 China lmtian@jlu.edu.cn; Advanced Institute of Materials Science, Changchun University of Technology Changchun 130012 P. R. China bingwei@ccut.edu.cn

## Abstract

Correction for ‘Exploring the antifouling effect of elastic deformation by DEM–CFD coupling simulation’ by Limei Tian *et al.*, *RSC Adv.*, 2019, **9**, 40855–40862, DOI: 10.1039/C9RA06761B.

The authors regret that Fig. 2e and f in the original article displayed incorrect images. The correct version of Fig. 2 is given below. These changes do not affect the overall conclusions of the article.

**Fig. 2:**
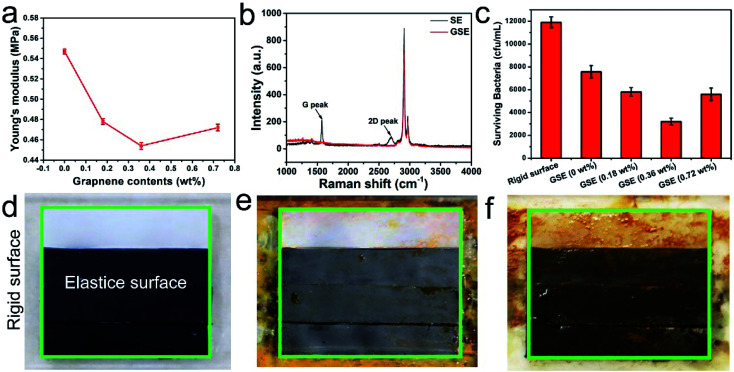
(a) The elastic modulus of pristine SE film and GSE film with different graphene content. (b) Raman spectra of the pristine SE and GSE films. (c) The surviving bacteria of *P. pantotrophus* incubated on rigid surface and antifouling surfaces. Representative digital images showed the rigid surface (outside the green border) and elastic surface (inside the green border) after incubated with *P. pantotrophus* for (d) 0 h, (e) 60 h and (f) 120 h in simulated marine environment. The graphene concentration of elastic surface is 0 wt%, 0.18 wt%, 0.36 wt% and 0.72 wt%, respectively.

The Royal Society of Chemistry apologises for these errors and any consequent inconvenience to authors and readers.

## Supplementary Material

